# Navigating the Digital Landscape: Embracing Innovation, Addressing Challenges, and Prioritizing Patient-Centric Care

**DOI:** 10.7759/cureus.58352

**Published:** 2024-04-15

**Authors:** Kanika Vats

**Affiliations:** 1 Department of Management, School of Commerce and Management, Om Sterling Global University, Hisar, IND; 2 Department of Healthcare Regulatory Affairs, Emirates Classification Society (TASNEEF), Abu Dhabi, ARE

**Keywords:** innovation, patient centric care, reputation, customer feedback, digitalization

## Abstract

In the digital era, healthcare customer feedback plays a pivotal role in shaping the reputation of healthcare organizations. The study explores how digital advancements are integrated into modern healthcare, offering both transformative insights and addressing the challenges they present. It investigates how technologies such as artificial intelligence (AI), digital platforms, and patient feedback systems impact patient care, operational efficiency, and customer satisfaction in healthcare settings. The study emphasizes the importance of balancing both capitalizing on the opportunities presented by innovations and addressing the inherent difficulties associated with digitalization in healthcare, underlining the need for a comprehensive approach to navigating the opportunities and challenges in healthcare digitalization.

AI is recognized for its role in reshaping value creation in healthcare, fostering collaboration among stakeholders, and improving patient care. Additionally, the study identifies key areas of research essential for effectively navigating the digital transformation in healthcare, including operational efficiency, patient-centric strategies, and organizational factors.

However, along with the potential benefits come challenges, such as the need for regulatory frameworks to validate new technologies and address privacy concerns surrounding patient data. Managing reputation and customer relationships in the digital sphere also emerges as critical for healthcare organizations.

In summary, the study underscores the importance of healthcare institutions prioritizing patient-centric care, adopting digital innovations, and adeptly navigating regulatory and ethical challenges. By doing so, they can enhance patient outcomes, and satisfaction, and drive innovation in today's dynamic healthcare landscape.

## Introduction and background

The article was originally presented as an abstract at the 2024 Emerging Trends in Engineering, Commerce, Management and Hospitality Management in the Digital Age for Sustainable Future international conference on March 16 and 17, 2024 in India.

The global healthcare system faces the monumental task of addressing medical, lifestyle, and personal health needs, straining healthcare resources [1]. To confront these challenges, empowering populations to self-manage their health through health innovations is crucial for enhancing well-being and alleviating the burden on healthcare resources. The past decade has witnessed significant technological progress in healthcare, marking a remarkable shift. The COVID-19 pandemic further accelerated the adoption of innovative technologies [2-3].

Digital transformation, an ongoing process, presents opportunities within the health sector, contingent upon the availability of infrastructure and training. Regulation (EU) 2021/694 defines digital transformation as the utilization of digital technologies to revolutionize businesses and services [4]. Digital technologies have been pivotal in addressing pandemics, ensuring uninterrupted connectivity despite geographical limitations. Digital health solutions enable direct interaction between healthcare professionals and patients, irrespective of location, facilitating patient self-management and bridging the gap between patients and providers [5].

The integration of innovative digital solutions in healthcare poses significant challenges, as it requires breaking down data and knowledge silos. This is especially crucial considering that digital technologies are expected to remain central to healthcare delivery in many instances [6-7]. However, it has also led to notable shifts in patient engagement. Historically authoritarian, healthcare interactions have transitioned toward collaborative engagement, driven by widespread smartphone and internet accessibility. This shift is evident in the digitization of healthcare, empowering patients to collect personalized health data, manage their healthcare remotely, and engage directly with providers [8].

Patient feedback plays a critical role in elevating healthcare quality and results, providing invaluable insights into patient experiences and areas needing focus. Integrating patient feedback allows healthcare providers to tailor services to align with patient preferences, nurturing patient-centered care and catalyzing system-wide enhancements. While navigating the digital era poses challenges such as managing negative feedback and ensuring data privacy and security, it also presents opportunities to leverage positive feedback to strengthen brand perception.

Siegel and King emphasize the pivotal role of artificial intelligence (AI) in revolutionizing healthcare [9-10]. AI has the potential to strengthen patient involvement, streamline claims processing, improve diagnostic precision, tailor healthcare to individual needs, and optimize resource allocation within hospitals.

The study sought to provide valuable insights into the transformative impact, challenges, and opportunities related to the importance of patient feedback in the digital era. Subsequently, it aimed to propose recommendations and future research prospects for healthcare organizations to effectively utilize digital innovations, thereby enhancing patient-centered care, improving operational efficiency, and fostering innovation in contemporary healthcare delivery.

## Review

Methods

A comprehensive literature search was conducted between January 2024 and February 2024, utilizing academic journals, conference proceedings, and reputable online databases available at Google Scholar and ResearchGate Platforms to gather relevant literature on digital innovations in healthcare. Carefully selected keywords were employed to capture all English-language publications published since 2020, with a focus on the integration of technologies such as AI, digital platforms, and patient feedback mechanisms in healthcare, emphasizing their impact on patient care, operational efficiency, customer satisfaction, and reputation management. The gathered data from 49 references underwent thorough analysis to identify key themes and insights related to the integration of digital innovations in modern healthcare settings. Exclusion criteria were applied to studies not in English, those with inaccessible full texts, or those solely focusing on patients' perspectives. Reviews addressing ethical and legal aspects of social media use were also omitted from the review process.

**Figure 1:**
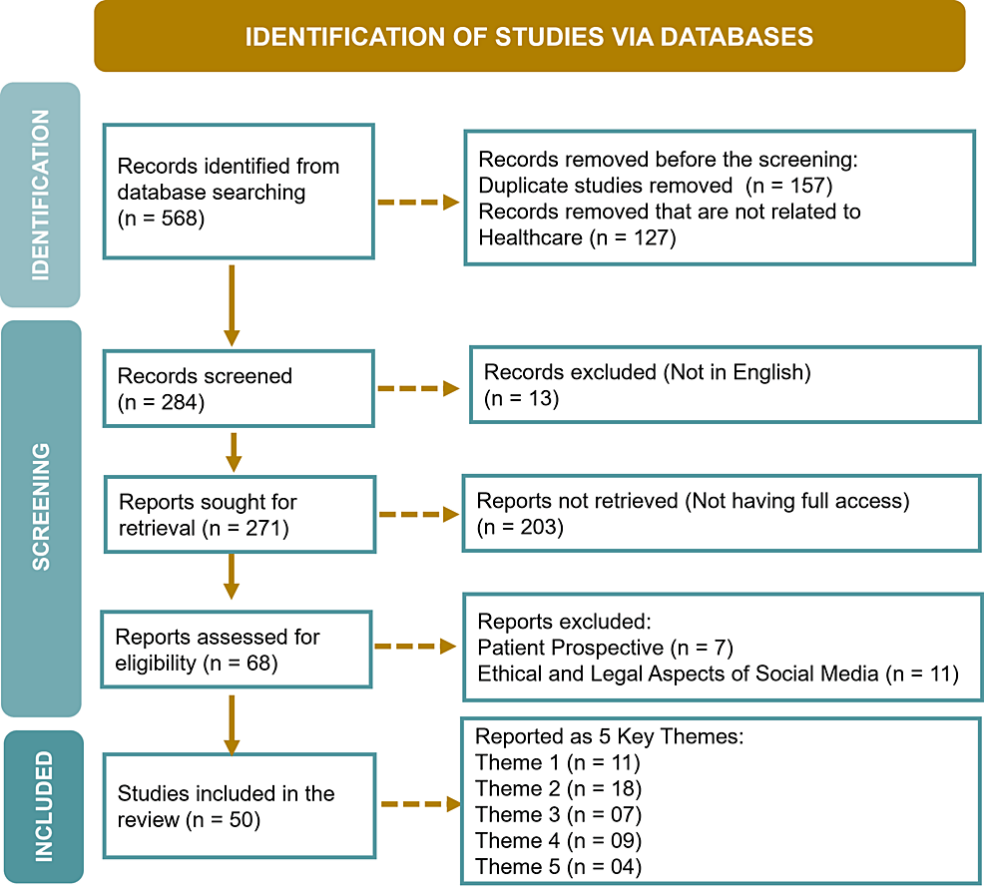
Study process flow and inclusion/exclusion criteria Credit: Image created by the author

Figure 1 outlines the study process, delineating the inclusion and exclusion criteria for this narrative review.

**Figure 2:**
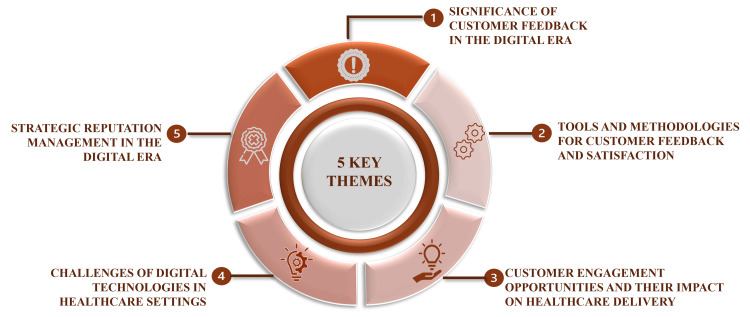
Five key themes illustrating literature review outcomes Credit: Image created by the author

The review is condensed into five key themes: the significance, tools and methodologies, opportunities, challenges, and reputation management to discuss the prospects concerning the integration of digital advancements and the importance of customer feedback in modern healthcare settings, with a view to enhancing patient-centric care (Figure 2).

Significance of customer feedback in the digital era

The review underscores the imperative role of customer satisfaction in the success of healthcare products and services. It collectively underscores the imperative for healthcare providers to adopt comprehensive customer relationship management (CRM) strategies that prioritize patient-centered care, employee engagement, and continuous quality improvement to accomplish exceptional patient outcomes and loyalty.

Baashar et al. highlight the benefits of CRM in healthcare for patient care and hospital efficiency across three areas: eCRM, system implementation, and adoption [11]. Moreover, Sundaram et al. have also emphasized that the use of patient-reported experience measures (PREMs) data plays a substantial role in improving health services quality [12].

Health team involvement, diverse feedback channels, and quality tools are deemed essential for leveraging patient feedback to enhance quality improvement efforts [13]. Endeshaw encouraged that internal employee perspectives in healthcare quality assessment are as equal as emphasizing patient-centric approaches hence, customized quality measurement frameworks are essential for each country and healthcare organization [14].

Khan et al. have further assessed the relationships between customer commitment, satisfaction, trust, and loyalty and found that CRM and corporate reputation positively impact customer loyalty. Furthermore, customer satisfaction mediates the relationship between CRM, corporate commitment, and customer loyalty [15].

Patients prefer sharing treatment experiences with doctors with higher medical quality and service attitude, especially in hospitals with a strong online reputation. Patients are more inclined to share experiences of doctors treating less severe diseases [16]. This was further accentuated by Umoke et al. in a study that underscores that patient satisfaction is a critical metric for assessing both the quality of care and the performance of healthcare facilities. Regular patient satisfaction assessments are also recommended for healthcare reform [17].

As per Abekah‐Nkrumah et al., there is a significant positive correlation between CRM, patient satisfaction, and patient loyalty, with patient satisfaction also correlating significantly with patient loyalty. Furthermore, factors such as education, health facility ownership, health insurance status, and gender do not significantly influence these relationships nor directly impact patient satisfaction and loyalty [18]. Zaman et al. also found that patient satisfaction impacts provider choice and medical outcomes and, therefore, may benefit the prioritization of improvement efforts [19]. A similar impact on customer satisfaction was assessed by Ali et al. affecting customer belief and profitability. Analysis reveals that privacy concerns significantly affect customer satisfaction and loyalty [20].

The quality of physician practice and hospital environment also has a significant impact on outpatient revisit intent, while for inpatients, physician practice and the kindness of medical staff directly influence revisit intent. Overall, delivering high-quality medical services and genuine patient care in all departments is essential for enhancing satisfaction and encouraging return visits from patients [21] as indicated by Woo and Choi.

Tools and methodologies for customer feedback and satisfaction

Various tools and methodologies are explored by various experts, including AI for value co-creation, digital platforms for patient engagement, process mining for evaluating customer journeys, and machine learning for sentiment analysis. These tools offer insights into customer behavior, organizational performance, and service quality in healthcare settings.

Boylan et al. indicated that online patient feedback presents a convenient and accessible means to capture healthcare experiences, though considerations for digital exclusion are important [22], whereas digital platforms hold promise for enhancing patient diagnosis, consultation, and treatment, but the lack of official regulations complicates the validation and approval of new health technologies as specified by Senbekov et al. [23].

Arias et al. underlined that using process mining methods in healthcare to analyze customer journeys provides notable benefits because of their efficiency. Scrutinizing healthcare procedures from the perspective of customer journey elements enables effective enhancement of the patient experience [24]. Also, engaging patients in tailoring digital feedback methods is key to integrating them into standard practices hence, Ong et al. proposed that implementing digital feedback must account for local settings, diverse patient populations, and leadership roles [25].

Shah et al. have introduced an innovative technique to assess online physician reviews (OPRs) through a blend of sentic computing methodologies (i.e., combining AI and semantic web strategies), that has offered significant insights to enhance service quality within healthcare institutions [26]. Whereas Schiavone et al. emphasized that economy-based platforms can improve customer experience and transform healthcare processes by showcasing potential innovations through a model that highlights value co-creation at individual, network, and community levels [27].

Libai et al. have found that AI is revolutionizing CRM into AI-CRM, significantly affecting customer acquisition, development, and retention processes. AI-CRM offers predictive capabilities for customer lifetime value, with substantial implications for businesses and regulatory frameworks [28]. Service robots are another alternative, as proposed by Lu et al., that can affect both customers and employees. Customers adjust their acceptance, and notice changes in robot traits and service quality, while employees see workload decrease but may experience reduced autonomy [29]. Holland et al. have further found that robots are beneficial for patients, healthcare staff, consumers, and organizations during the COVID-19 crisis by focusing on improved sanitation, logistics for patients and supplies, minimizing errors, and monitoring patients remotely to improve hospital operations, efficiency, and resource allocation [30].

Cobelli and Chiarini stressed the significance of professionals adopting multichannel communication like mHealth and supply strategies and underscored the pharmacy industry's acknowledgment of the importance of such approaches for ensuring customer satisfaction and loyalty [31]. Farsi has further mentioned that effective healthcare involves integrating various dimensions, such as combining healthcare with social media and other communication channels. Finding the optimal blend of digital and traditional healthcare is crucial for success [32].

Data collected from patient-reported outcome measures (PROMs) and PREMs are essential for a patient-centered healthcare model, collected via interviews and consensus sessions with users to create guidelines for their integration into clinical practices, as indicated by Wolff et al. [33].

Ren and Ma revealed that the COVID-19 crisis has accelerated the use of online medical services for their convenience and infection control benefits. Governments are endorsing virtual consultations to maintain healthcare access during restrictions, while doctors are driven by financial incentives to engage in web-based platforms for consultations [34].

AI's influence on healthcare is evident through its application in diagnosing illnesses, treating patients, and managing hospital operations as a co-creation in B2B markets, notably in healthcare, fostering collaboration and enhancing patient care. However, successful integration requires careful planning and execution to maximize its advantages in healthcare [35-36]. Jain et al. have further indicated another methodology of machine learning that plays a crucial role in analyzing consumer sentiment in healthcare, highlighting their potential to inform management strategies and guide future research paths [37].

Alshurideh has specified that electronic CRM positively influences service quality and hence suggests enhancing the implementation of electronic customer relations management by reassessing website design, ensuring ease of use for patients, and incorporating search functionality into the website [38]. This was further underlined by Bez et al., who stated that digital feedback platforms may enhance hospital operations by analyzing patient feedback, identifying concerns, suggesting improvements, and fostering innovation, thereby boosting business intelligence and supporting digital transformation [39].

Customer engagement opportunities and their impact on healthcare delivery

The review also encapsulates the versatile landscape of online customer engagement and its profound implications for healthcare delivery. From revealing consumer psychology to harnessing digital innovations for operational enhancement, it provides a comprehensive overview of the evolving dynamics shaping modern healthcare paradigms.

Rasool et al. highlighted that the surge in online customer engagement has transformed consumers into collaborative creators of value and indicated three primary research focuses emerge: comprehending customer psychology, enhancing digital interaction, and utilizing it for increased profitability and loyalty [40].

The involvement of professionals in creating understandable formats and offering assistance in interpreting data has been pointed out by Hancock et al. [41], which will support identifying new feedback methods and data formats. Gustafsson et al. have found a significant gap in research concerning missed care from patients' viewpoints, yet it's crucial. Patients frequently mention lacking essential care, communication, and punctuality. Instances of missed care remain a major hurdle for everyone involved [42].

Another opportunity was highlighted by Patrício et al. by proposing a human-centered service design approach that can leverage technology for people-centered care, drive innovative solutions for healthcare change, and address the complexity of healthcare systems through integrated care, hence promoting advancement through interdisciplinary collaboration, methodological innovation, and theoretical grounding [43].

Knowledge management (KM) was displayed as equal importance by Migdadi for leveraging opportunities that encompass knowledge acquisition, diffusion, application, knowledge from customers, knowledge about customers, and knowledge for customers, influencing the success of CRM. This, in turn, affects innovation capabilities (IC), with KM impacting IC through the success of CRM [44].

This was followed by the identification of five key areas of digital healthcare research by Kraus et al. which are operational efficiency, patient-focused approaches, organizational aspects, workforce methods, and socio-economic factors by proposing a model illustrating how technology improves operational efficiency for healthcare providers [45].

Ramsey et al. found online patient feedback helpful for organizations to learn and improve. How organizations work together, collaborate across different fields, and focus on learning are crucial factors affecting the usefulness of this feedback [46].

Challenges of digital technologies in healthcare settings

Despite the opportunities presented by digital innovations, the review also highlights several challenges, including health data integrity, health misinformation spread through social media, and barriers to integrating electronic patient-reported outcomes, that persist and require proactive solutions.

Wong et al. have highlighted the requirement of comprehensive tactics to enhance both patient satisfaction and the quality of care provided by proposing a multi-faceted approach to tackle both individual and organizational elements that tend to yield superior results [47].

The importance of anonymous patient feedback was highlighted by Locock et al., by acknowledging that despite successful personalization and customization efforts, some staff members felt uncomfortable with it. There were concerns about the validity of feedback because patients chose to remain anonymous. Staff members who are used to direct patient interactions may need support in handling anonymous feedback and understanding its power dynamics [48].

Yang et al. indicated that both a doctor's professional standing and patient feedback have a significant role in a patient's selection of a doctor. Moreover, the seriousness of the patient's condition affects this dynamic: for severe illnesses, professional status matters more, while for less severe ones, feedback has greater influence. Additionally, the study revealed the severity of illness reduced the interchangeability of professional status and feedback in influencing patient choice [49].

Additionally, the increasing utilization of social media brings forth significant user data, yet simultaneously presents privacy concerns. Efforts to detect weaknesses and address privacy hazards have prompted the development of anonymization methods, as indicated by Beigi and Liu [50].

Nguyen et al. have emphasized that resource allocation must be prioritized by healthcare providers and the government to improve service quality. Practitioners should focus on building their social presence and enhancing online services to strengthen connections with patients [51].

Later, Zarour et al. replicated a concern within the healthcare sector for maintaining the integrity of health data in the digital age, leading to potential problems like fraud and inadequate treatment, and proposed solutions to tackle these issues effectively with proactive steps i.e., blockchain, are essential to ensure the security of healthcare information [52].

Suarez-Lledo and Alvarez-Galvez have drawn attention to the use of social media platforms that have become a significant channel for spreading health misinformation, with Twitter being particularly notable for its role in both framing and disseminating such content, drawn attention by. Additionally, platforms like Facebook, Instagram, and YouTube also contributed significantly to the spread of health misinformation [53].

Another set of challenges was enlightened by Glenwright et al. by identifying factors that either assist or impede the integration of electronic PROMs and PREMs within healthcare environments, offering significant perspectives to enhance patient-reported outcomes [54].

Recently this year Khan et al. underscored the challenges such as technological issues, barriers at the practice level, and personal limitations that impede the full utilization of online services. Despite recognizing the convenience of digital options, Di-Facto survey participants emphasized the importance of in-person doctor visits [55].

Strategic reputation management in the digital era

Maintaining effective reputation management becomes crucial in public administration. This is essential for fostering strong organizational ties and effectively addressing both internal and external challenges.

Bustos has mentioned that reputation management in public administration is vital for fostering strong organizational relationships and effectively addressing internal and external challenges. Maintaining a positive reputation not only reinforces fundamental elements such as brand, trust, and identity but also secures organizational legitimacy and success [56].

The Control, Access, Responsive, Engagement (CARE) model proposed by Chaudhri et al. highlights the dualistic nature of social-mediated communication. This model indicates that healthcare professional responses are crucial for managing reputation implications in the constantly active realm of social media [57].

Another strategy proposed by Huang et al., combining online and offline services in e-healthcare can lead to increased online demand and enhanced professional reputation, providing insights for better design and management of e-health platforms [58].

Last, Olaimat et al. have further stressed upholding a positive image by businesses and stakeholders in today's digital era. Leveraging social media can help effectively manage reputations. Utilizing public relations methods, alongside closely monitored protocols, underscores the significance of skilled communication strategies in this endeavor [59].

Recommendations

Healthcare organizations can effectively harness the transformative potential of digital innovations to improve patient-centric care, enhance operational efficiency, and drive innovation in modern healthcare delivery by adopting recommendations such as effective investment in AI and digital technologies for better patient care and operational efficiency, developing robust regulatory frameworks to validate new health technologies and safeguard patient data, ensuring strict adherence to privacy regulations and employing encryption for patient data security, providing adequate training for healthcare professionals on using digital tools effectively, actively involving patients in designing and implementing digital solutions to improve usability and satisfaction, fostering interdisciplinary collaboration among stakeholders for innovation and problem-solving, promoting a culture of continuous improvement by gathering feedback and making iterative changes, encouraging knowledge sharing among peers to disseminate best practices and lessons learned, and developing strategic long-term plans considering technological advancements and evolving patient needs.

Future scope

A longitudinal study is recommended to assess the long-term impact of digital innovations in healthcare. This could involve tracking the implementation and utilization of various technologies over an extended period to evaluate their effectiveness, sustainability, and evolving outcomes on patient care, operational efficiency, and customer satisfaction. Additionally, exploring the socioeconomic implications, ethical considerations, and regulatory challenges associated with the sustained integration of digital innovations in healthcare could provide valuable insights for future decision-making and policy development.

## Conclusions

Effective healthcare requires integrating various dimensions, including combining healthcare with social media and other communication channels. While digital platforms promise to enhance patient diagnosis, consultation, and treatment, the lack of official regulations complicates the validation and approval of new health technologies. Digital feedback platforms offer valuable insights for hospitals, facilitating organizational learning and quality improvement efforts. Reputation management, CRM, and corporate reputation positively impact customer loyalty and satisfaction in healthcare settings. Privacy concerns significantly affect customer satisfaction and loyalty, underscoring the importance of addressing privacy issues in healthcare services.

In conclusion, healthcare organizations must prioritize patient-centered care, embrace digital innovations, and address challenges related to privacy, data integrity, and regulatory compliance to succeed in the evolving landscape of healthcare delivery. The long-term impact of utilizing digitalization should also be assessed to support evidence-based decision-making. By doing so, they can not only improve patient outcomes and satisfaction but also drive organizational growth and innovation in the healthcare sector.
